# Nomograms for predicting the overall survival of patients with cerebellar glioma: an analysis of the surveillance epidemiology and end results (SEER) database

**DOI:** 10.1038/s41598-021-98960-3

**Published:** 2021-09-29

**Authors:** Jie Li, Wobin Huang, Jiajing Chen, Zhuhui Li, Bocong Liu, Peng Wang, Jun Zhang

**Affiliations:** Neurosurgery, Dalang Hospital, Dongguan, 523770 Guangdong China

**Keywords:** Cancer, Surgical oncology

## Abstract

At present, our understanding of cerebellar glioma is still insufficient. This study collected information on patients in the SEER database to identify the predictive factors for patients with cerebellar glioma. Data from patients with cerebellar glioma diagnosed from 1975 to 2018 were retrieved from the Surveillance Epidemiology and End Results Database. We randomly divided the patients into a training group and a validation group, established a nomogram based on the training group, and used the validation group data to verify the clinical value of the model. A total of 508 patients were included in this study. Multivariate analysis was performed based on the data before randomization, and the results showed that the patient's age, WHO grade, histological type, and extent were significantly correlated with the survival rate. The C-index of the OS nomograms of the training cohort was 0.909 (95% CI, (0.880–0.938)) and 0.932 (95% CI, (0.889–0.975)) in the validation group. The calibration curve of OS for 3 and 5 years showed that there was good consistency between the actual survival probability and the predicted survival probability. For patients with cerebellar glioma, the age at diagnosis, WHO grade of the glioma, histological type, and extension are the four factors that most strongly affect the overall survival outcomes. Furthermore, our model may be a useful tool for predicting OS in these patients.

## Introduction

Gliomas are common intracranial tumours, but gliomas that occur in the cerebellum are very rare, especially in adults^1^. Because of its rarity, there have been few previous studies in this area, so it is necessary to conduct research in this field. Regardless of the patient’s age, histology, or pathology, cerebellar gliomas are serious neurological tumours that usually manifest as life-threatening complications, such as compression of the cerebellum and adjacent brainstem or obstruction of the cerebrospinal fluid circulation pathway, resulting in increased intracranial pressure, which leads to serious consequences and even threatens the life of the patient. Some researchers have reported that patients with cerebellar glioblastoma have a poor prognosis, with a survival period of three to seven months^2,3^, while others have observed that cerebellar glioblastoma and brain glioblastoma have similar survival times^4,5^. In this article, we collected information on patients with cerebellar glioma from the SEER database, explored the factors that affect the overall survival time of cerebellar glioma, and constructed a clinical prognostic model of cerebellar glioma to provide risk assessment for clinical patients.

## Methods

### Study population

**Figure 1:**
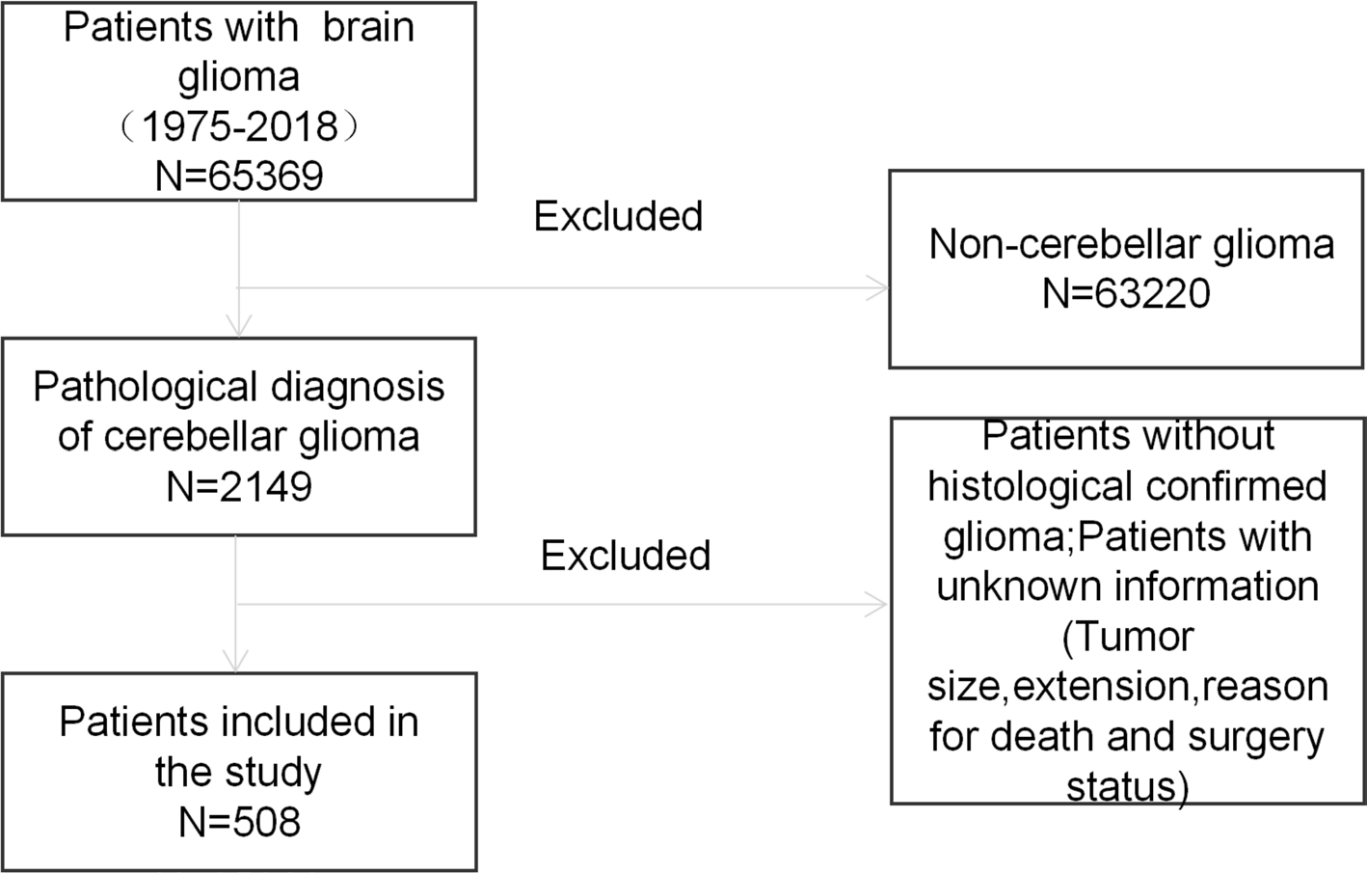
Flowchart of cerebellum glioma patient selection.

The data of patients with cerebellar glioma diagnosed from 1975 to 2018 were retrieved from the Surveillance Epidemiology and End Results Database. The Surveillance Epidemiology and End Results Database collects *information* about patients’ baseline *characteristics* and information on tumours, such as histological characteristics, WHO grade, surgery, and patients’ vital status, in 9 registries within the United States. By using SEER*Stat software (Version 8.3.9), we retrieved data from 65,369 patients in total. The inclusion criteria included (1) first primary cerebellar glioma; (2) ICD-O-3 site codes limited to the cerebellum (C71.6); and (3) detailed information on sex, race, tumour size, WHO grade, surgery information, histological characteristics, pathological diagnosis, and patient vital status. The exclusion criteria included (1) patients without histologically confirmed glioma and (2) patients with unknown information, such as tumour size, extension, reason for death and surgery status. A flowchart of cerebellar glioma patient selection is shown in Fig. 1.

### Included covariates

The data included for analysis were as follows: age (≤ 20 years old was defined as the paediatric group, and > 20 years old was defined as the adult group), race (white, black and other) and sex. The cerebellar glioma characteristic data obtained for analysis were tumour size (size ≤ 3.0 cm, size > 3.0 cm) and tumour extension (cerebellum, brainstem, ventricle and other). Histological characteristics (Pilocytic astrocytoma, anaplastic astrocytoma, ependymoma, glioblastoma, and other). WHO grade (WHO grades III and IV were assigned to high grade, WHO grades I and II were assigned to low grade). The extent of tumour resection (local excision/biopsy, STR, GTR, and resection lobe of brain).

### Statistical analyses

The variables associated with OS in this study included age, race, sex, year at diagnosis, WHO grade, tumour size, histological type, and extent of surgery. *We used the* Kaplan–Meier method to assess the overall survival rate and the log-rank test to evaluate the differences between survival curves. Then, we used univariate and multivariate analyses to determine the effect of prognostic factors on OS. Both multivariate and univariate Cox proportional hazard models were used to calculate HRs (hazard ratios) and 95% CIs (confidence intervals). *P* < 0.05 was considered statistically significant. The population was randomly divided into a validation group (*n* = 152) and a training group (*n* = 356). Then, we used the RMS package in R version 4.1.0 (http://www.r-project.org/) to build a nomogram model to calculate the risk score according to the formula. Calibration curves and Harrell’s concordance index (C-index) were used to estimate the accuracy and consistency of the nomogram between the predicted and actual OS rates.

## Results

### Patient population and baseline characteristics

A total of 508 patients were included in this study. Demographic information, surgery information and tumour characteristics are described in Table 1.

**Table 1:** Patient demographics, tumor characteristics and treatment of 508 patients with histologically confirmed cerebellar gliomas.

Characteristics	ALL	Local excision/biopsy	STR	GTR	Resection lobe of brain	*P*-value
Population size	508	24 (4.7%)	152 (29.9%)	174 (34.3%)	158 (31.1%)	
**Age**						< 0.001
< 20	290 (57.1%)	5 (20.8%)	66 (43.4%)	121 (69.5%)	98 (62.0%)	
20–60	151 (29.7%)	4 (16.7%)	62 (40.8%)	39 (22.4%)	46 (29.1%)	
> 60	67 (13.2%)	15 (62.5%)	24 (15.8%)	14 (8.0%)	14 (8.9%)	
**Race**						0.221
White	396 (78.0%)	17 (70.8%)	122 (80.3%)	131 (75.3%)	126 (79.7%)	
Black	58 (11.4%)	1 (4.2%)	15 (9.9%)	25 (14.4%)	17 (10.8%)	
Other	54 (10.6%)	6 (25.0%)	15 (9.9%)	18 (10.3%)	15 (9.5%)	
**Sex**						0.346
Female	252 (49.6%)	13 (54.2%)	83 (54.6%)	78 (44.8%)	78 (49.4%)	
Male	256 (50.4%)	11 (45.8%)	69 (45.4%)	96 (55.2%)	80 (50.6%)	
**Year**						< 0.001
2011–2015	252 (49.6%)	16 (66.7%)	91 (59.9%)	142 (81.6%)	3 (1.9%)	
2004–2010	256 (50.4%)	8 (33.3%)	61 (40.1%)	32 (18.4%)	155 (98.1%)	
**Hist**						< 0.001
Glioblastoma	67 (13.2%)	3 (12.5%)	31 (20.4%)	17 (9.8%)	16 (10.1%)	
Ependymoma	36 (7.1%)	1 (4.2%)	9 (5.9%)	15 (8.6%)	11 (7.0%)	
Anaplastic ast	29 (5.7%)	9 (37.5%)	10 (6.6%)	6 (3.4%)	4 (2.5%)	
Pilocytic ast	325 (64.0%)	4 (16.7%)	86 (56.6%)	129 (74.1%)	106 (67.1%)	
Other	51 (10.0%)	7 (29.2%)	16 (10.5%)	7 (4.0%)	21 (13.3%)	
**WHO grade**						< 0.001
Low	52 (10.2%)	1 (4.2%)	16 (10.5%)	7 (4.0%)	28 (17.7%)	
High	67 (13,2%)	11 (45.8%)	25 (16.4%)	18 (10.3%)	13 (8.2%)	
Unknow	389 (76.6%)	12 (50.0%)	111 (73.0%)	149 (85.6%)	117 (74.1%)	
**Extension**						0.213
Cerebellum	381 (75.0%)	17 (70.8%)	105 (69.1%)	137 (78.7%)	122 (77.2%)	
Brainstem	59 (11.6%)	4 (16.7%)	20 (13.2%)	16 (9.2%)	19 (12.0%)	
Ventricular	31 (6.1%)	1 (4.2%)	9 (5.9%)	9 (5.2%)	12 (7.6%)	
Other	37 (7.3%)	2 (8.3%)	18 (11.8%)	12 (6.9%)	5 (3.2%)	
**Tumor size**						< 0.001
< 3 cm	161 (31.7%)	16 (66.7%)	55 (36.2%)	42 (24.1%)	48 (30.4%)	
> 3 cm	347 (68.3%)	8 (33.3%)	97 (63.8%)	132 (75.9%)	110 (69.6%)	
**Vital status**						< 0.001
Alive	129 (25.4%)	8 (33.3%)	103 (67.8%)	143 (82.2%)	125 (79.1%)	
Dead	379 (74.6%)	16 (66.7%)	49 (32.2%)	31 (17.8%)	33 (20.9%)	

*GTR* gross total resection, *STR* subtotal resection, *HIST* Histologic type, *Anaplastic ast* anaplastic astrocytoma, *Pilocytic ast* pilocytic astrocytoma.

The average age of our study was 25.5 years old, and the average survival time of patients was 73.8 months. In our study, white people accounted for 78.0% of the population, whereas 58 patients (11.4%) were black, and 54 patients (10.6%) were defined as “other race”. Patients diagnosed from 2011–2015 were almost unchanged compared to those diagnosed from 2004–2010 (*n* = 252 (49.6%), vs. *n* = 256 (50.4%). From the perspective of tumour histology, the majority of tumours were pilocytic astrocytomas (325, 64.0%). Sixty-seven (13.2%) patients had glioblastoma, 29 (5.7%) had anaplastic astrocytoma, 36 (7.1%) had ependymoma, and 51 (10.0%) were defined as the other group. In terms of tumour WHO grade, 52 (10.2%) were assigned to the low group, 67 (13.2%) were assigned to the high group, and 389 (76.6%) were defined as the other group according to the most recent CBTRUS. A total of 75.0% of tumours were confined to the cerebellum, 59 tumours (11.6%) extended to the brainstem, and 31 tumours (6.1%) extended to the ventricle. A total of 24 (4.7%) patients received Local excision/biopsy only, 152 (29.9%) patients treated with received subtotal resection (STR), 174 (34.3%) patients underwent gross total resection (GTR), and 158 (31.1%) patients had a lobe of the brain resected. The overall survival rate of the GTR group was the highest (*P* < 0.001).

### Prognostic factors of OS

Univariate analysis was used to identify significant characteristics in Table 2.

**Table 2:** Univariate and multivariate analysis of overall survival.

Characteristics	Univariate analysis HR (95% CI)	*P*	Multivariate analysis HR (95%CI)	*P*
**Age**				
< 20	0.03 (0.02–0.06)	** < 0.001**	0.25 (0.13–0.49)	** < 0.001**
20–60	0.21 (0.15–0.31)	** < 0.001**	0.35 (0.23–0.53)	** < 0.001**
> 60	Reference		Reference	
**Race**				
White	1.11 (0.62–1.99)	0.72	0.76 (0.41–1.41)	0.39
Black	Reference		Reference	
Other	2.22 (1.12–4.40)	**0.02**	1.01 (0.47–2.16)	0.97
**Sex**				
Female	Reference			
Male	1.16 (0.82–1.64)	0.40		
**Year**				
2004–2010	0.96 (0.68–1.36)	0.80		
2011–2015	Reference			
**Hist**				
Glioblastoma	1.93 (1.19–3.12)	**0.007**	4.92 (2.55–9.46)	** < 0.001**
Ependymoma	0.23 (0.11–0.47)	** < 0.001**	0.78 (0.33–1.84)	0.57
Anaplastic astrocytoma	Reference		Reference	
Pilocytic astrocytoma	0.02 (0.01–0.04)	** < 0.001**	0.119(0.04–0.29)	** < 0.001**
Other	0.34 (0.19–0.62)	** < 0.001**	1.06(0.50–2.24)	0.87
**WHO grade**				
Low	0.08 (0.03–0.19)	** < 0.001**	0.30 (0.12–0.79)	**0.015**
High	Reference		Reference	
Unknow	0.14 (0.09–0.20)	** < 0.001**	0.43 (0.27–0.68)	** < 0.001**
**Extension**				
Cerebellum	0.45 (0.28–0.71)	** < 0.001**	0.60 (0.37–0.97)	**0.04**
Brainstem	Reference		Reference	
Ventricular	0.78 (0.37–1.65)	0.52	1.19 (0.57–2.58)	0.66
Other	1.40 (0.76–2.57)	0.28	1.00 (0.53–1.91)	0.99
**Tumor size**				
< 3 cm	Reference			
> 3 cm	0.78 (0.55–1.12)	0.18		
**Surgery**				
Local excision/biopsy	6.23 (3.40–11.41)	** < 0.001**	2.18 (1.10–4.29)	**0.02**
STR	2.02 (1.28–3.16)	**0.002**	1.10 (0.68–1.77)	0.70
GTR	Reference		Reference	
Resection lobe of brain	1.13 (0.69–1.84)	0.64	1.05(0.63–1.77)	0.84

Bold indicates *P* < 0.05.

**Figure 2:**
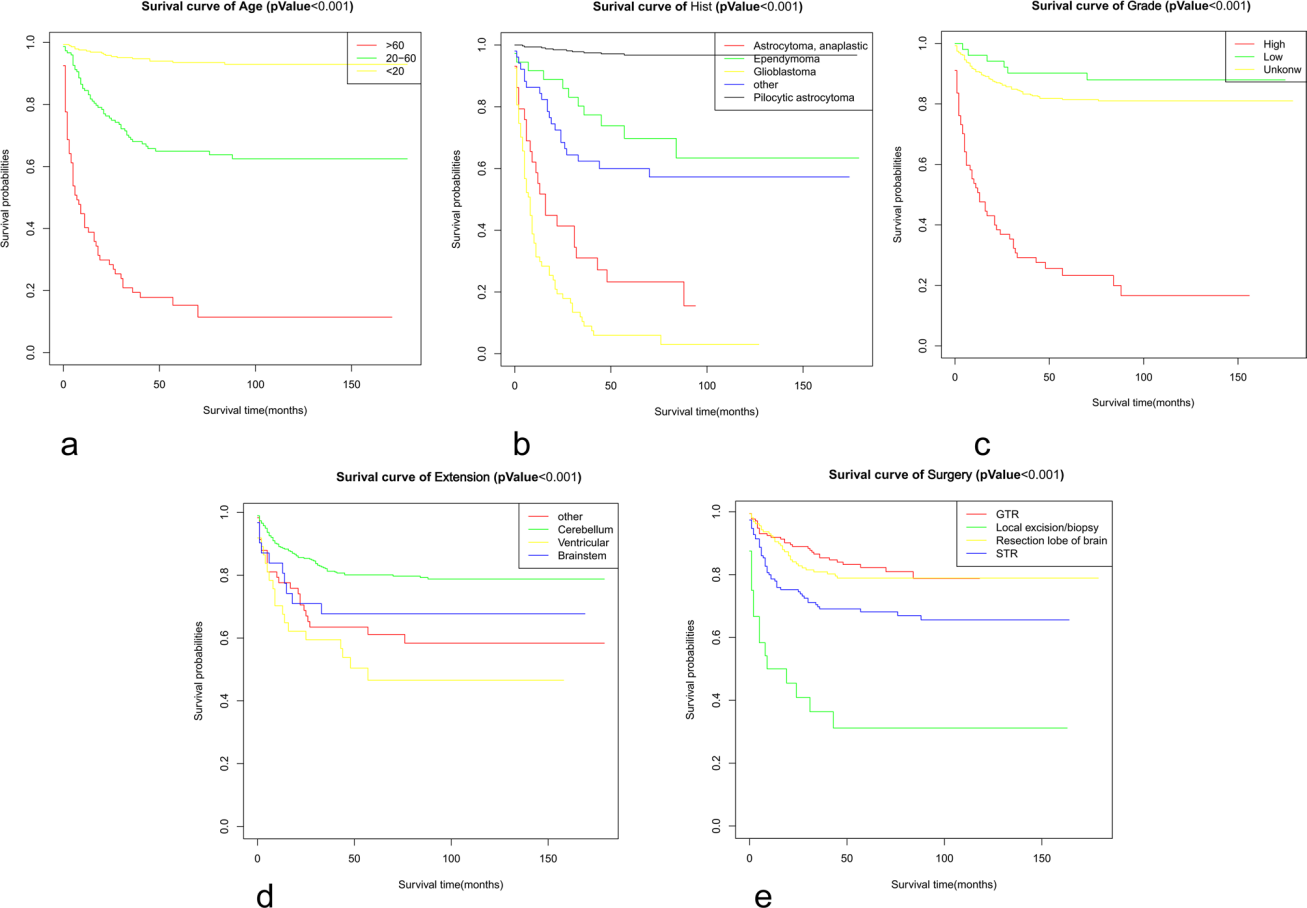
Kaplan–Meier curves for patients with cerebellum glioma by different variates. (**a**) Age, (**b**) Histology, (**c**) WHO Grade, (**d**) Extension, (**e**) Surgery.

**Figure 3:**
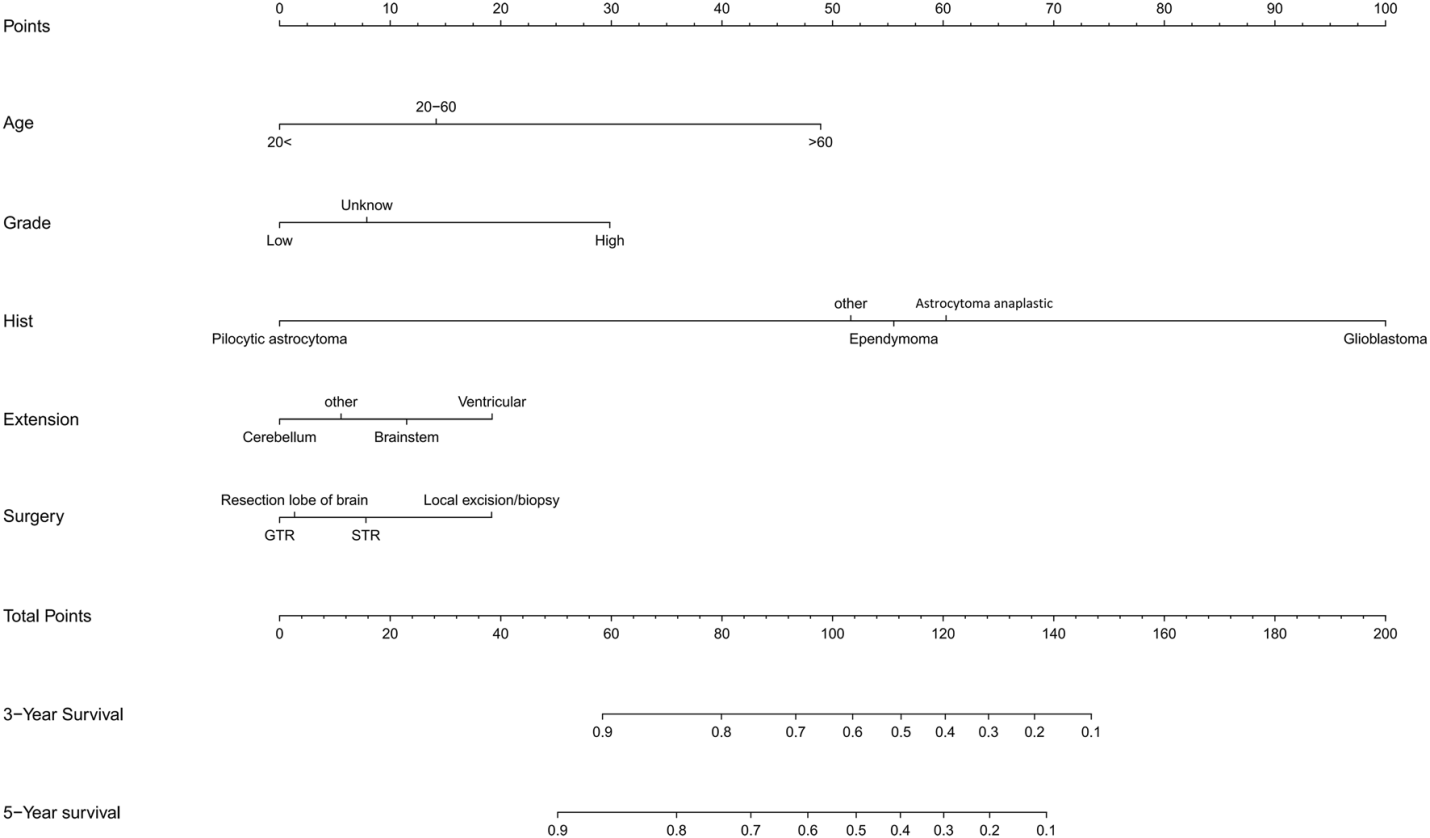
Nomogram for overall survival rate based on training group.

**Figure 4:**
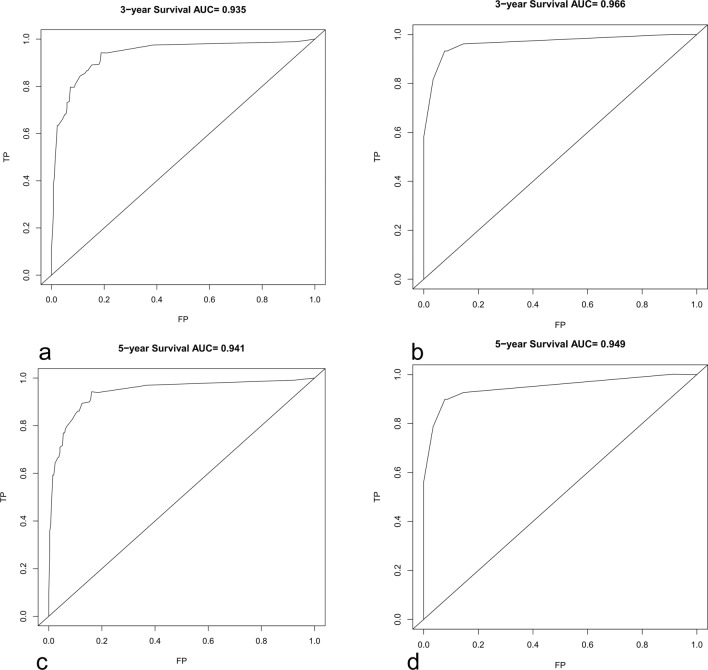
Time-dependent ROC curve and areas under ROC curve. Areas under ROC curve of 3-year overall survival rates in training group (**a**) and validation group (**b**). Areas under ROC curve of 5-year overall survival rates in training group (**c**) and validation group (**d**).

**Figure 5:**
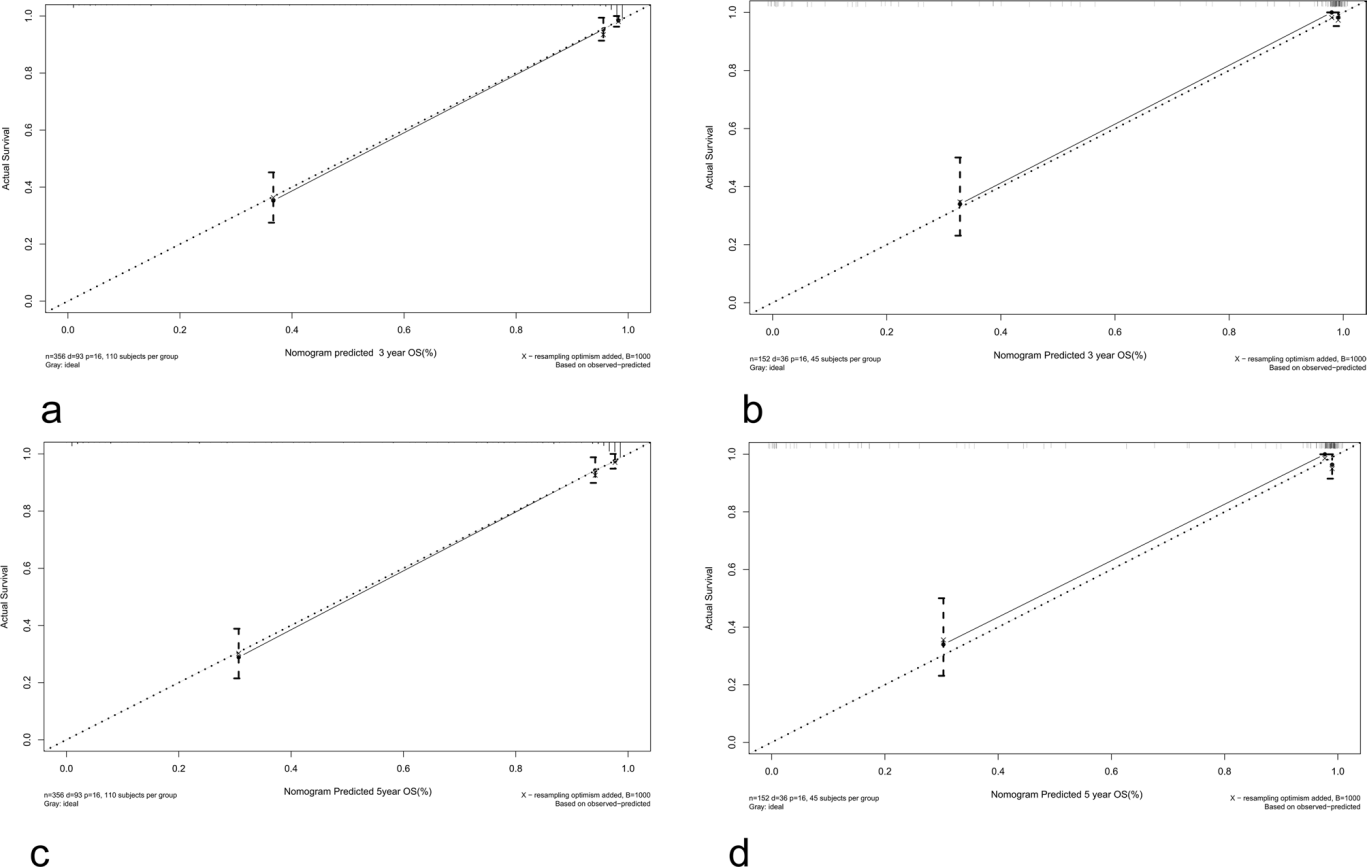
Internal calibration plots of 3-year overall survival rates in training group (**a**) and validation group (**b**) Internal calibration plots of 5-year overall survival rates in training group (**c**) and validation group (**d**).

Kaplan–Meier curves of age, histologic type, tumour WHO grade, tumour extension, and surgery were painted to compare the overall survival of cerebellar glioma patients, as shown in Fig. 2.The results showed that age (*P* < 0.001), histology type (*P* < 0.001), WHO grade (*P* < 0.001), and surgery (*P* < 0.001) were related to the overall survival of the patients. Then, we randomly divided the data into a validation group and a training group. There were 356 people in the training group and 152 people in the validation group. There was no significant difference between the two groups (Table 3). Next, the nomogram model was built based on the five characteristics of the training group: age, race, extension, tumour grade, histological type, and surgery, as shown in Fig. 3. The 3-year and 5-year overall survival rates were evaluated by a nomogram to compute the corresponding score. The C-index (receiver operating curve) and internal calibration curve were plotted to verify the accuracy and discrimination of the model. The C-index of overall survival rate prediction was 0.909 (95% CI, (0.880–0.938)) and 0.932 (95% CI, (0.889–0.975)), respectively. The receiver operating curve (ROC) and area under the curve (AUC) were plotted. The area under the curve values of the 3-year and 5-year overall survival rates in the training group were 0.935 and 0.941, respectively, and those in the validation group were 0.966 and 0.949, respectively (Fig. 4), which suggested that the predictive model was effective and accurate. The calibration curve of the overall survival rate at 3 years and 5 years showed that there was excellent consistency between the predicted survival probability and the actual survival probability (Fig. 5).

**Table 3:** Training and validation of cerebellum glioma patient.

Characteristics	All *n* (%)	Training *n* (%)	Validation *n* (%)	*p*
**Age**				0.586
< 20	290 (57.1%)	198	92	
20–60	151 (29.7%)	109	42	
> 60	67 (13.2%)	49	18	
**Race**				0.405
White	396 (78.0%)	273	123	
Black	58 (11.4%)	45	13	
Other	54 (10.6%)	38	16	
**Sex**				0.373
Female	252 (49.6%)	172	80	
Male	256 (50.4%)	184	72	
**Year**				0.615
2004–2010	252 (49.6%)	182	74	
2011–2015	256 (50.4%)	174	78	
**Hist**				0.396
Glioblastoma	67 (13.2%)	50	17	
Ependymoma	36 (7.1%)	27	9	
Anaplastic astrocytoma	29 (5.7%)	17	12	
Pilocytic astrocytoma	325 (64.0%)	225	100	
Other	51 (10.0%)	37	14	
**WHO grade**				0.206
Low	52 (10.2%)	42	10	
High	67 (13.2%)	46	21	
Unknow	389 (76.6%)	268	121	0.300
**Extension**				
Cerebellum	381 (75.0%)	268	113	
Brainstem	59 (11.6%)	41	18	
Ventricular	31 (6.1%)	25	6	
Other	37 (7.3%)	22	15	
**Tumor size**				0.651
< 3 cm	161 (31.7%)	115	46	
> 3 cm	347 (68.3%)	241	106	
**Surgery**				0.957
Local excision/biopsy	24 (4.7%)	17	7	
STR	152 (29.9%)	109	43	
GTR	174 (34.3%)	120	54	
Resection lobe of brain	158 (31.1%)	110	48	
**Status**				0.563
Alive	129 (25.4%)	263	116	
Dead	379 (74.6%)	93	36

## Discussion

Central nervous system (CNS) tumours account for the majority of malignant and benign neoplasms prevalent across the entire age spectrum^6^. Although the incidence of central nervous system tumours is higher in adults, it is also the main cause of death in children^7^. Cerebellar glioma is a common type of central nervous system tumour in children but is relatively rare in adults^1^. Since the incidence of cerebellar glioma is not high, few studies have focused on this area. Central nervous system tumours, especially tumours of the cerebellum and brainstem, cause the greatest proportion of deaths (37.9% and 16.6%, respectively)^8^. Patients with cerebellar glioma often present with the symptom of intracranial hypertension and some cerebellar symptoms, such as ataxia in walking, unstable standing, dizziness, mental disorders and other symptoms that seriously affect the quality of daily life^2,9–13^. Our analysis of patients with cerebellar glioma demonstrated that age is an important factor affecting the prognosis, such that younger patients are more likely to have a better prognosis. The difference in the prognosis of children and adults may be caused by differences in the molecular mechanism of tumours, but our study did not include the study of molecular mechanisms because there is no relevant information about molecular mechanisms in the SEER database. Ranjith Babu et al. found that patients older than 40 years had poor outcomes, and patients aged over 65 years had the worst survival rate (4 versus 12 months, *p* < 0.0001) compared to younger patients^14^. Our study found that the prognosis of patients with cerebellar glioma is independent of sex. In terms of race, univariate analysis showed that there was no difference in the prognosis of white and black patients, and the prognosis of the rest of the population was worse, but the difference disappeared after multivariate analysis. *Zhuoyi Liu* reported that whether in univariate analysis or multivariate analysis, the prognosis of tumours has nothing to do with sex and race^15^. Our study found that cerebellar glioblastoma had the worst prognosis, followed by anaplastic astrocytoma, and pilocytic astrocytoma had the best prognosis. These differences were statistically significant regardless of univariate analysis or multivariate analysis. Glioblastoma has a high degree of malignancy, its growth rate is blocky, the boundary is not clear, and the operation is difficult to complete, which is the main reason for the poor prognosis. Xingwang Zhou et al. demonstrated that oligodendroglioma, anaplastic glioma and glioblastoma were risk factors for early mortality in children and pointed out that glioblastoma increases early childhood mortality^16^. Ankush Chandra et al. found a very meaningful study: tumours > 40 mm were associated with decreased survival for patients with supratentorial glioblastoma^17^. However, in our study, the prognosis of cerebellar glioma had little to do with the size of the tumour. This may be because there is no obvious correlation between the size and the degree of malignancy of the tumour. In this study, we found that the higher the WHO grade of the tumour, the worse the prognosis, and the lower the patient’s median survival time and overall survival rate. Adams et al. found that the prognosis of high-grade gliomas was much worse than that of low-grade gliomas^18^. Some scholars found that the higher the grade of glioma, the higher the activity level and the higher the tumour angiogenesis^19^. Additionally, malignant high-grade gliomas are diffuse and infiltrating lesions that often infiltrate some important peripheral functional areas, which seriously affect the quality of life of patients^20^. In terms of the extension of the tumour, tumours confined to the cerebellum have the best prognosis, and the prognosis is poor after expansion to the brainstem. It may be that after the tumour has expanded to the brainstem, it is difficult to completely remove because the brainstem is the core part of the brain. Weber et al. found that the extent of surgery and brainstem involvement were associated with poorer survival^14^. The conclusion is roughly the same as ours. Regarding the extent of tumour resection, univariate analysis showed that patients with total tumour resection had the best prognosis and the highest overall survival rate. Univariate analysis showed that patients with total tumour resection had the best prognosis and the highest overall survival rate. However, in a multivariate analysis, the overall survival rate of the patient had little to do with the degree of tumour resection. Our research also has many limitations. First, the data available in SEER database are observational. The allocation of subjects is arbitrary and lacks randomization. Second, we do not have the authority to obtain information about radiotherapy and chemotherapy. Currently, chemotherapy is considered to be the standard treatment for patients with the following diseases: high-grade glioma in the United States. In addition, other important factors are not easily available in the SEER database, which includes the patient’s preoperative symptoms, imaging data, family financial status, postoperative neurological function, and postoperative quality of life. Our research is a retrospective clinical study, with the general limitations of retrospective studies. Therefore, it is necessary to conduct high-quality prospective studies to verify our conclusions.

## Conclusion

Our study is a large-scale population-based study of cerebellar glioma. It directly compares factors such as age, sex, tumour size, histological type, grade, and resection and found that young age, pilocytic astrocytoma, low-grade tumours, and confined tumours can significantly prolong the survival time of patients with cerebellar glioma, while cerebellar glioblastoma can significantly reduce the survival time of patients. Our study provides very useful information for further research on cerebellar glioma.
